# Supplementary material for: Effects of an integrated intervention on schistosomiasis prevalence in a rural area of Tanzania
Source: PLoS Negl Trop Dis. 2025 Jul 2;19(7):e0013215. doi: 10.1371/journal.pntd.0013215 (PMC12221010; doi:10.1371/journal.pntd.0013215)
Supplement: S1 Text — (DOCX) [file pntd.0013215.s001.docx]

S1 Text : Questionnaire set

| **School name** |  | **Child name:** |
| --- | --- | --- |
| **District name:** |  | **Child ID:** |
| Ecological zones | Based on proximity to water bodies |  |
|  | 1.Near and less than 1KM |  |
|  | 2.Medium-1-5KM |  |
|  | 3.Geater than 5KM |  |
| **S/no** | **Questions** | **Responses** |
| 1 | What is your sex/gender  *Jinsia yako ni ipi?* | 1. Male *(Kiume)* |
|  |  | 1. Female (*Kike)* |
| 2 | What is your age?  *Una umri gani?* |  |
| 3 | How many people are living in your parent house?  *Watu wangapi wanaishi katika nyumba ya wazazi wako?* |  |
| 4 | What is the level of education of your mother?  *Ni kipi kiwango cha elimu cha mama yako?* | 1. Illiterate/did not go to school [Hajasoma/ hakwenda shule] |
|  |  | 1. Did not complete primary school [Hakumaliza Elimu ya msingi] |
|  |  | 1. Completed primary school [*Amehitimu elimu ya msingi]* |
|  |  | 1. Higher education (secondary, college, university) *Elimu ya juu-sekondari, chuo, chuo kikuu)* |
| 5 | What is the level of education of your father?  *Ni kipi kiwango cha elimu cha baba yako?* | 1. Illiterate/did not go to school [Hajasoma/ hakwenda shule] |
|  |  | 1. Did not complete primary school [Hakumaliza Elimu ya msingi] |
|  |  | 1. Completed primary school [Amehitimu elimu ya msingi] |
|  |  | 1. Higher education (secondary, college, university) [*Elimu ya juu-sekondari, chuo, chuo kikuu]* |
| 6 | What is your mother’s occupation?  *Ni ipi kazi ya mama yako?* |  |
| 7 | What is your father’s occupation?  *Ni ipi kazi ya baba yako?* |  |
| **Sanitation and hygiene question (*Maswali kuhusu usafi wa mwili na mazingira*)** | | |
| 8 | *Where do you prefer to defecate or urinate?*  *Unapendelea kujisaidia/kunya wapi au kukojoa wapi?* | 1. Anywhere in the bushes or near the lake/river (Mahala popote kwenye vichaka au karibu na ziwa/mto*)* |
|  |  | 1. In toilets (*katika vyoo)* |
| 9 | Do you have toilet at home?  *Je, una choo nyumbani*? | 1. No (Hapana) |
|  |  | 1. Yes (Ndiyo) |
| 10 | If Yes at 9, which type of toilet of toilet is present at your home?  *Kama ndiyo katika swali la 9, je ni aina gani ya choo kilichopo nyumbani kwako?* | 1. Pit latrine (Choo cha shimo) |
|  |  | 1. Pour/Flush toilet (Choo cha kumwaga maji /cha kuflashi) |
|  |  | 1. No toilet at home (Hakuna choo nyumbani) |
| 11 | Do you have toilet at school? Je, mna choo shuleni? | 1. No (*Hapana*) |
|  |  | 1. Yes (*Ndiyo*) |
| 12 | Do you regularly use toilet at school and at home?  *Je, huwa unatumia choo mara kwa mara shuleni na nyumbani?* | 1. No (*Hapana*) |
|  |  | 1. Yes (*Ndiyo*) |
| 13 | If NO at 12, where do you defecate or urinate at school or at home?  Kama hapana katika swali la 12, Je huwa unakunya au kukojoa wapi shuleni na nyumbani? | 1. Anywhere in the bushes or near the lake/river (*Mahala popote kwenye vichaka au karibu na ziwa/mto*) |
|  |  | 1. In toilets (Chooni) |
| 14 | What is the source of water at home? (Ni kipi chanzo cha maji nyumbani?) | 1. Lake (Ziwa) |
|  |  | 1. Rivers/streams (Mito/ mifereji) |
|  |  | 1. Portable/piped water   (*Maji ya bomba / maji ya chupa*) |
|  |  | 1. Protected spring (Chemu chemu/kisima iliyotunzwa) |
|  |  | 1. Unprotected spring [Chemu chemu/kisima ya asili isiyotunzwa] |
|  |  | 1. Hand pumped wells [Kisima kirefu chenye pampu] |
| 15 | What type of water sources do you use for drinking at home | 1. Portable/piped water (maji ya bomba) |
|  |  | 1. River (mtoni) |
|  |  | 1. Lake (ziwani) |
|  |  | 1. Open well/spring (Kisima/chemu chemu ya wazi) |
|  |  | 1. Closed well (kisima kilichofungwa na kina pampu) |
| 16 | How often do you contact water bodies (river, streams, ponds, canal) in a week? Ni mara ngapi unakwenda bwawani, mtoni, kisimani kwa wiki? | 1. None |
|  |  | 1. Once a week |
|  |  | 1. Two times a week (mara mbili kwa wiki) |
|  |  | 1. Three times a week |
|  |  | 1. More than three times a week |
| **Behavioural risks (personal hygiene) Tabia hatarishi ( Usafi binafsi)** | | |
| 17 | Do you wash your hands after defecating or urinating/ do you wash your hands after visiting toilet?  *Je, huwa unanawa mikono yako baada ya kunya au kukojoa/ huwa unanawa mikono yako baada ya kutumia choo?* | 1. No (*Hapana*) |
|  |  | 1. Yes (*Ndiyo*) |
| 18 | Do you wash your hands prior to eating foods/meals [*Huwa unanawa mikono kabla ya kula chakula/ milo?]* | 1. No (*Hapana*) |
|  |  | 1. Yes (*Ndiyo*) |
| 19 | Do you often play with soil?  *Je, huwa unachezea udongo mara kwa mara?* | 1. No (*Hapana*) |
|  |  | 1. Yes (*Ndiyo*) |
| 20 | Do you wash your hands after playing with soil?  *Je, huwa unanawa mikono yako baada ya kuchezea udongo*? | 1. No (*Hapana*) |
|  |  | 1. Yes (*Ndiyo*) |
| 21 | Do you wear shoes regularly?  *Je, huwa unavaa viatu mara kwa mara*? | 1. No (*Hapana*) |
|  |  | 1. Yes (*Ndiyo*) |
| 20 | How many times do you wear shoes per week?  *Je, unavaa viatu mara ngapi kwa wiki?* | 1. Once per week (*Mara moja kwa wiki*) |
|  |  | 1. At least twice week (*Angalau mara mbili kwa wiki*) |
|  |  | 1. All days of the week (*Siku zote za wiki*) |
| 21 | Do you wash fruits or vegetables before eating?  *Je, unaosha matunda na mbogamboga kabla ya kula*? | 1. No (Hapana) |
|  |  | 1. Yes (*Ndiyo*) |
| 22 | Do you cut your nails regularly?  Je huwa unakata kucha zako mara kwa mara? | 1. No (*Hapana*) |
|  |  | 1. Yes (*Ndiyo*) |
| 23 | If YES at 21, which method do you use to cut your nails?  *Kama ndiyo katika swali la 21, Je unatumia njia gani kukata kucha zako*? | 1. Using my teeth (*Kutumia meno*) |
|  |  | 1. Using razor blade/nail cutter/knife (*kwa kutumia wembe/ kifaa cha kukatia kucha/kisu*) |
| 24 | Do you have animals (dog, cat)/livestock (pigs, cattle, goats, sheep, chicken) at home?  *Je, mna wanyama (mbwa, paka)/ mifugo (nguruwe, ng’ombe, mbuzi, kondoo, kuku) nyumbani*? | 1. No (*Hapana*) |
|  |  | 1. Yes (*Ndiyo*) |
| 25 | Do you regularly contact these animals?  *Je, huwa unawashika hawa wanyama mara kwa mara?* | 1. No (*Hapana*) |
|  |  | 1. Yes (*Ndiyo*) |
| 26 | Have you ever received anti-helminthic at school?  *Je, umeshawahi kupokea dawa za minyoo shuleni?* | 1. No (*Hapana*) |
|  |  | 1. Yes (*Ndiyo*) |
| 27 | Have you ever received anti-helminthic at home?  *Je, umeshawahi kupokea dawa za minyoo nyumbani?* | 1. No (*Hapana*) |
|  |  | 1. Yes (*Ndiyo*) |
