# Supplementary material for: Effects of an integrated intervention on schistosomiasis prevalence in a rural area of Tanzania
Source: PLoS Negl Trop Dis. 2025 Jul 2;19(7):e0013215. doi: 10.1371/journal.pntd.0013215 (PMC12221010; doi:10.1371/journal.pntd.0013215)
Supplement: S1 Table — (DOCX) [file pntd.0013215.s002.docx]

S1 Table. Effects on intermediate outcomes among male school-aged children

|  | SMDA only  (active control) | | SMDA plus CMDA | | Fully integrated model  (SMDA, CMDA, CLTS, CVA) | | | |
| --- | --- | --- | --- | --- | --- | --- | --- | --- |
| Survey round | Baseline | Endline | Baseline | Endline | Baseline | Endline | Ref. SMDA | Ref. CMDA |
| Water contact  behavior (3 times or more)  n/N  (%) | 17/30  (56.7%) | 10/29  (34.5%) | 90  /221  (40.7%) | 139  /221  (62.9%) | 72  /85  (84.7%) | 52  /92  (56.5%) | -6.0% (-33.8%,21.8%) | -50.4%***(-66.0%,-34.7%) |
| Praziquantel | 30/30 (100.0%) | 26/28  (92.9%) | 144  /221  (65.2%) | 204  /223  (91.5%) | 52  /85  (61.2%) | 85  /86  (98.8%) | NA | 11.3%  (-1.5%, 24.2%) |
| Latrine at home | No (0/30, 0%)  Unimproved (29/30, 96.7%)  Improved (0/30, 0%) | No (0/29, 0%)  Unimproved (24/29, 82.8%)  Improved (0/29, 0%) | No (1/254, .03%)  Unimproved (199 /254, 78.3%)  Improved (4/254, 1.6%) | No (0/226, 0%)  Unimproved (146 /226, 64.6%)  Improved (69 /226, 30.5%) | No (0/85, 0%)  Unimproved (81 /85, 95.3%)  Improved (1/85, 1.2%) | No (0/92, 0%)  Unimproved (54 /92, 58.7%)  Improved (34 /92, 37.0%) | NA | NA |
| Latrine at school | 24/30 (80%) | 29/29 (100%) | 201 /221 (91.0%) | 226 /226 (100%) | 67/85 (78.8%) | 92/92 (100%) | NA | NA |
| Use latrine at home to defecate & urinate | 30/30 (83.6%) | 14/29 (100%) | 200 /221 (91.6%) | 152 /226 (64.5%) | 80 /85 (95.0%) | 87 /91 (94.4%) | NA | 24.7%*** (15.0%, 34.5%) |
| Wash hands after using latrine | 27/30 (90%) | 17/28 (60.7%) | 110 /221 (49.8%) | 125 /223 (56.1%) | 43/85 (50.6%) | 82/92 (89.1%) | 67.8%*** (43.4%, 92.2%) | 32.3%*** (16.7%, 47.7%) |
| Wash hands before having meals | 30/30 (100%) | 28/28 (100%) | 213 /221 (96.4%) | 222 /225 (98.7%) | 85/85 (100%) | 92/92 (100%) | NA | -2.3% (-5.2%, 0.6%) |

*Abbreviations: NA not applicable

*p-value: p<.05 *, p<.01 **, p<.001 ***
