# Supplementary material for: Effects of an integrated intervention on schistosomiasis prevalence in a rural area of Tanzania
Source: PLoS Negl Trop Dis. 2025 Jul 2;19(7):e0013215. doi: 10.1371/journal.pntd.0013215 (PMC12221010; doi:10.1371/journal.pntd.0013215)
Supplement: S2 Table — (DOCX) [file pntd.0013215.s003.docx]

S2 Table. Effects of interventions on schistosomiasis prevalence (male school-aged children)

|  | | SMDA only  (active control) | | SMDA plus CMDA | | Fully integrated model  (SMDA, CMDA, CLTS, CVA) | | |
| --- | --- | --- | --- | --- | --- | --- | --- | --- |
| Survey round | | Baseline | Endline | Baseline | Endline | Baseline | Endline | |
| n/N  (prevalence, %) | | 4/30  (13.3%) | 0/29  (0.0%) | 74/254  (29.1%) | 6/226  (2.6%) | 37/85  (43.5%) | 2/92  (2.2%) |  |
| OR (95% CI) | | 1 | 1 | 0.983 (-0.104, 2.070) | 15.964 (-3898.0, 3930.0) | 1.612** (0.475, 2.748) | 16.759 (-6436.3, 6469.8) |  |
| Risk difference  (95%  CI) | Ref. SMDA |  |  | 15.8%* (2.4%, 29.2%) | NA | 30.2%*** (14.1%, 46.3%) | 2.2%  (-0.8%, 5.2%) |  |
|  | Ref. Baseline |  | NA |  | -26.5%*** (-32.4%, -20.5%) |  | -41.4%*** (-52.3%, -30.4%) |  |
|  | DiD |  |  |  | NA |  | NA | -14.9%* (-27.4%, -2.4%) |

*Abbreviations: OR odds ratio, CI confidence interval, NA not applicable, SMDA school mass drug administration, CMDA community mass drug administration, DiD difference-in-difference, CLTS community-led total sanitation, CVA community voice and action

*p-value: p<.05 *, p<.01 **, p<.001 ***
