# Supplementary material for: Effects of an integrated intervention on schistosomiasis prevalence in a rural area of Tanzania
Source: PLoS Negl Trop Dis. 2025 Jul 2;19(7):e0013215. doi: 10.1371/journal.pntd.0013215 (PMC12221010; doi:10.1371/journal.pntd.0013215)
Supplement: S3 Table — (DOCX) [file pntd.0013215.s004.docx]

S3 Table. Effects on intermediate outcomes among female school-aged children

|  | SMDA only  (active control) | | SMDA plus CMDA | | Fully integrated model  (SMDA, CMDA, CLTS, CVA) | | | |
| --- | --- | --- | --- | --- | --- | --- | --- | --- |
| Survey round | Baseline | Endline | Baseline | Endline | Baseline | Endline | Ref. SMDA | Ref. CMDA |
| Water contact  behavior (3 times or more)  n/N  (%) | 27/31  (87.1%) | 4/31  (12.9%) | 84  /206  (40.8%) | 156  /244  (63.9%) | 74  /95  (77.9%) | 34  /88  (38.6%) | 34.9% ** (13.7%,56.2%) | -62.4%*** (-78.4%,-46.5%) |
| Praziquantel | 30/31 (98.4%) | 30/30  (96.6%) | 133  /206  (64.9%) | 215  /249  (88.8%) | 68/95  (66.7%) | 77/85  (95.9%) | 18.0%**  (5.6%,30.2%) | -0.6%  (-13.8%,12.6%) |
| Latrine at home | No (0/31, 0%)  Unimproved (31/31, 100%)  Improved (0/31, 0%) | No (0/29, 0%)  Unimproved (26/29, 89.7%)  Improved (3/29, 10.3%) | No (3/241,1.2%)  Unimproved (180 /241, 74.7%)  Improved (3/241, 1.2%) | No (0/254, 0%)  Unimproved (182 /254, 71.7%)  Improved (54 /254, 21.3%) | No (0/96, 0%)  Unimproved (91/96, 94.8%)  Improved (1/96, 1.0%) | No (0/88, 0%)  Unimproved (56/88, 63.6%)  Improved (28/88, 31.8%) | NA | 11.0%  (-0.8%, 22.7%) |
| Latrine at school | 27/31 (90.3%) | 31/31 (100%) | 175 /206 (85.0%) | 254 /254 (100%) | 80/95 (84.2%) | 88/88 (100%) | 2.9% (-11.0%,16.7%) | 0.7% (-8.1%, 9.6%) |
| Use latrine at home to defecate & urinate | 30/31 (96.8%) | 30/31 (96.8%) | 191 /206 (92.7%) | 157 /253 (62.1%) | 91/95 (95.8%) | 83/88 (94.3%) | 1.5% (-12.3%, 9.3%) | 29.2%*** (19.8%, 38.6%) |
| Wash hands after using latrine | 29/31 (93.5%) | 30/30 (100%) | 108 /206 (52.4%) | 143 /249 (57.4%) | 54/95 (56.8%) | 82/88 (93.2%) | 67.8%*** (43.4%, 92.2%) | 31.3%*** (16.8%, 45.9%) |
| Wash hands before having meals | 31/31 (100%) | 30/30 (100%) | 205 /206 (99.5%) | 252 /252 (100%) | 94/95 (98.9%) | 87/88 (98.9%) | NA | -0.6% (-3.7%, 2.6%) |

*Abbreviations: NA not applicable

*p-value: p<.05 *, p<.01 **, p<.001 ***
