# Supplementary material for: Effects of an integrated intervention on schistosomiasis prevalence in a rural area of Tanzania
Source: PLoS Negl Trop Dis. 2025 Jul 2;19(7):e0013215. doi: 10.1371/journal.pntd.0013215 (PMC12221010; doi:10.1371/journal.pntd.0013215)
Supplement: S4 Table — (DOCX) [file pntd.0013215.s005.docx]

S4 Table. Effects of interventions on schistosomiasis prevalence (female school-aged children)

|  | | SMDA only  (active control) | | SMDA plus CMDA | | Fully integrated model  (SMDA, CMDA, CLTS, CVA) | | |
| --- | --- | --- | --- | --- | --- | --- | --- | --- |
| Survey round | | Baseline | Endline | Baseline | Endline | Baseline | Endline | |
| n/N  (prevalence, %) | | 3/31  (9.7%) | 0/31  (0.0%) | 61/241  (25.3%) | 4/254  (1.6%) | 31/96  (32.3%) | 3/88  (3.4%) |  |
| OR (95% CI) | | 1 | 1 | 1.152  (-0.074, 2.377) | 15.431 (-3770.2, 3801.1) | 1.493* (0.228, 2.758) | 17.222 (-6224.2, 6258.7) |  |
| Risk difference  (95%  CI) | Ref. SMDA |  |  | 15.6%*** (3.9%, 27.4%) | NA | 22.6%** (8.6%, 36.6%) | NA |  |
|  | Ref. Baseline |  | NA |  | -23.7%*** (-29.4%, -18.0%) |  | -28.9%*** (-39.0%, -18.8%) |  |
|  | DiD |  |  |  | -13.7%* (-24.5%,  -2.9%) |  | NA | -5.1% (-16.7%, 6.4%) |

*Abbreviations: OR odds ratio, CI confidence interval, NA not applicable, SMDA school mass drug administration, CMDA community mass drug administration, DiD difference-in-difference, CLTS community-led total sanitation, CVA community voice and action

*p-value: p<.05 *, p<.01 **, p<.001 ***
