# Supplementary material for: Effects of an integrated intervention on schistosomiasis prevalence in a rural area of Tanzania
Source: PLoS Negl Trop Dis. 2025 Jul 2;19(7):e0013215. doi: 10.1371/journal.pntd.0013215 (PMC12221010; doi:10.1371/journal.pntd.0013215)
Supplement: Attachment — Submitted filename: Review.docx [file pntd.0013215.s008.docx]

Title: Effects of an integrated intervention on schistosomiasis prevalence in a rural area of Tanzania

Manuscript ID: PNTD-D-24-01456

The quasi-experimental field study assessed the effectiveness of integrated control efforts, both in school-aged children and adult community members, against *S. mansoni* and *S. haematobium* in an endemic region in Tanzania. The results showed that integrated efforts including MDA in children and adults, sanitation improvements, and community health education decreased the risk of schistosomiasis infection and the behavioral changes that favor infection control. The paper has listed several weaknesses of the study conducted that may limit the interpretation and generalizability of the results. Nevertheless, it provided proof of the superior effectiveness of integrated control measures targeting all members of an endemic community through diverse methods that augment the pharmacological intervention.

The paper is generally well-written and may be publishable in its submitted form. However, several issues need to be addressed. These are the following:

1. In the methods section, it would be beneficial if the authors could provide a subsection on the primary and supplementary outcomes that were measured. Regarding this issue, the methods section both Kato-katz and urine sedimentation techniques to detect schistosomiasis cases. The authors are asked to clarify how a positive sample was determined (e.g., were both tests considered for it to be positive, or either one being positive is enough). Also, how data on both species of *Schistosoma* species handled should be clarified because the paper does not separate them.
2. In the discussion section, it is appreciated that similar studies looking at *S. mansoni* and *S. haematobium* were cited as comparators, especially those conducted in other endemic African regions. It is also recommended that the authors consider similar studies on schistosomiasis control in Asia. Particularly, the large-scale integrative efforts that have been implemented by the Chinese government against schistosomiasis japonica with significant success in bringing down infections within the Yangtze River endemic foci. In contrast, heavy reliance on MDA as the primary control effort that led to minimal success can be lifted from the literature on schistosomiasis from the Philippines. Likewise, the success of integrative control efforts against opisthorchiasis in Northeastern Thailand can also be looked into to provide further insight. Increasing the diversity of references discussed would be beneficial to the study and also to the local governmental bodies that will be basing their decisions based on the findings of the paper.

I find that the paper will be considered publishable if these issues are addressed. I thank the researchers for their valuable research that is beneficial in the control of schistosomiasis in Tanzania.
