# Supplementary material for: Effects of an integrated intervention on schistosomiasis prevalence in a rural area of Tanzania
Source: PLoS Negl Trop Dis. 2025 Jul 2;19(7):e0013215. doi: 10.1371/journal.pntd.0013215 (PMC12221010; doi:10.1371/journal.pntd.0013215)
Supplement: Attachment — Submitted filename: PNTD-D-24-01456-comments.docx [file pntd.0013215.s009.docx]

**Reviewer comments;**

The manuscript focuses on the effectiveness of integrated interventions in controlling schistosomiasis in rural Tanzania. The study design is innovative, combining school-based mass drug administration (SMDA), community-based MDA (CMDA), and integrated approaches with community-led sanitation and health education. The results demonstrate the potential benefits of integrated disease control strategies, with statistical analysis using multilevel logistic regression. These findings are valuable for policymakers and public health practitioners in endemic areas. However, several aspects of the manuscript could be improved for clarity and overall quality:

**Major comments;**

- The study design raises a critical question regarding the timing of sample collection and intervention. The fecal samples were collected in September 2020, and the interventions (SMDA, CMDA, and integrated interventions) were implemented nearly two years later, in June and July 2022. This means the parasites were monitored 3–4 months after diagnosis and treatment. It is unclear whether the observed effects reflect the immediate short-term impact of treatment or the prevention of new infections during this period. The authors should clarify whether the intervention's effectiveness was assessed for short-term outcomes or its ability to prevent reinfections in the study participants over time.

- Consider incorporating GIS-based maps to visually represent the spatial distribution of schistosomiasis prevalence before and after the interventions. These maps could enhance the reader's understanding of the geographical impact and effectiveness of the integrated intervention strategy.

**Minor comments;**

- The tables presenting prevalence and behavioral outcomes (e.g., Tables 2 and 4) could be simplified by focusing on key statistically significant results. Consider adding concise bar graphs or heat maps for better clarity. Some of the detailed data can be moved to a supplementary file.

- The manuscript would benefit from more detail on why this particular study area was selected. What are the specific epidemiological challenges, such as high prevalence, ecological factors, or gaps in previous interventions, that make it an appropriate setting for addressing the research question? Providing this context would help strengthen the study's rationale and relevance to broader public health challenges.

- The quasi-experimental design risks selection bias due to non-randomized groups, affecting the robustness of the results. Please include a discussion on this limitation in the study.

- Although gender is mentioned as a variable, the study does not explore its specific impact on intervention outcomes. Given the potential differences in water-contact behaviors and sanitation practices between males and females, it would be valuable to analyze and discuss gender-disaggregated data. This would help determine whether the interventions were equally effective for boys and girls, as well as for men and women.
